# A Review of Inkjet Printed Graphene and Carbon Nanotubes Based Gas Sensors

**DOI:** 10.3390/s20195642

**Published:** 2020-10-02

**Authors:** Twinkle Pandhi, Ashita Chandnani, Harish Subbaraman, David Estrada

**Affiliations:** 1 Micron School of Materials Science and Engineering, Boise State University, Boise, ID 83725, USA; twinklepandhi@u.boisestate.edu; 2 Department of Electrical and Computer Engineering, Boise State University, Boise, ID 83725, USA; AshitaChandnani@u.boisestate.edu (A.C.); harishsubbaraman@boisestate.edu (H.S.); 3 Idaho National Laboratory, Idaho Falls, ID 83402, USA

**Keywords:** graphene, carbon nanotubes, inkjet printing, additive manufacturing, gas sensors, flexible electronics

## Abstract

Graphene and carbon nanotube (CNT)-based gas/vapor sensors have gained much traction for numerous applications over the last decade due to their excellent sensing performance at ambient conditions. Inkjet printing various forms of graphene (reduced graphene oxide or modified graphene) and CNT (single-wall nanotubes (SWNTs) or multiwall nanotubes (MWNTs)) nanomaterials allows fabrication onto flexible substrates which enable gas sensing applications in flexible electronics. This review focuses on their recent developments and provides an overview of the state-of-the-art in inkjet printing of graphene and CNT based sensors targeting gases, such as NO_2_, Cl_2_, CO_2_, NH_3_, and organic vapors. Moreover, this review presents the current enhancements and challenges of printing CNT and graphene-based gas/vapor sensors, the role of defects, and advanced printing techniques using these nanomaterials, while highlighting challenges in reliability and reproducibility. The future potential and outlook of this rapidly growing research are analyzed as well.

## 1. Introduction

Early detection of gases and harmful vapors has become increasingly important in many fields, such as environmental pollution monitoring [1,2,3], national defense [4,5], industrial emission monitoring [1,6,7], and medical diagnosis [5,8]. The fundamental sensing mechanism focuses on how well the gas sensors respond to the changes in the local environment. Furthermore, the need for flexible and portable gas sensors that show high sensitivity and selectively to gas analytes in real-time is growing significantly [9,10]. The emergence of materials such as carbon nanotubes (CNTs) and two-dimensional (2D) materials (e.g., graphene and MoS_2_) have shown great potential in targeting chemical and biological analytes, as well as in monitoring state variables, such as temperature, humidity, and pressure [11,12,13]. The exemplary electrical and structural properties of these materials allow for the design of highly sensitive and selective systems while also limiting the cost, weight, and energy consumption of electronic devices.

Graphene is an attractive sensing material for printed and flexible gas sensing device development due to its flexible nature, high surface to volume ratio, unique band structure, and high electrochemical activity at defect sites [12,14,15,16,17]. Due to its high specific surface area, high carrier mobility, and tunable crystal defect density, graphene has shown extraordinary properties and created tremendous breakthroughs in related electronics applications, particularly when it comes to trace gas/vapor sensing [18,19,20,21,22,23]. Synthesis of graphene by chemical vapor deposition (CVD), segregation by heat treatment of silicon carbide, and liquid/chemical solvent-based exfoliation are currently areas of intense research [24,25,26,27,28,29,30,31]. Among these, solvent exfoliation is highly compatible with printable graphene ink formulation. Moreover, the synthesis of graphene oxide (GO) is first achieved by liquid exfoliation, following the Hummers method [32,33]. The introduction of carboxylic and carbonyl groups at the edge of the graphene sheets allows graphene to readily disperse in water. However, the disadvantage of introducing these groups is that the active layer becomes electrically insulating despite several attempts by researchers to reduce GO (rGO) [34]. Inkjet printing of rGO based gas/vapor sensors has been reported by several groups, which we will discuss further in this review [35,36,37,38,39,40].

Carbon nanotubes (CNTs) are another widely used material for gas sensing due to their unique electrical and mechanical properties [41]. They possess a very high surface area to volume ratio and very high sensitivity towards target analytes at room temperature [7,42]. Target analytes transfer charge upon adsorption on the nanotube sidewalls or at the junctions, which leads to changes in the conductance of the CNT network. Depending upon the density of the CNT mats used for performing detection, the charge transfer leads to changes in the conductance of the CNT network. This is the key sensing mechanism for CNT gas sensors [43,44]. CNTs are of two types: single-walled (SWNTs) and multi-walled (MWNTs). SWNTs are analogous to a single sheet of graphene rolled up with about a nanometer diameter while MWNTs are concentric graphene rolls with diameters on the order of hundreds of nanometers [41]. CNTs are synthesized by arc discharge [45], pulsed laser deposition [46], and chemical vapor deposition [47], which introduce different defect densities, and hence varying electrical and mechanical properties [41,48]. CNT synthesis techniques typically produce both metallic and semiconducting nanotubes which can be separated by density gradient ultracentrifugation (DGU) [49,50], The separated CNTs can then be dispersed in a solution to be printed by inkjet printing, allowing for rapid prototyping of printed gas sensors. Of the many challenges to printing carbon nanotubes inks, the predominant ones relate to the dispersion of CNTs in solvents and elimination of CNT bundles [51,52]. Functionalization of CNTs with various materials that change the chemical structure and enhance the sensing performance has allowed researchers to solve some of the dispersion-related limitations of pristine CNTs [53,54]. Inkjet printing of CNT-based inks for gas sensing applications has been reported by several groups [53,55], which we will further discuss in this paper.

An ideal gas sensor needs to provide the following features: (i) high sensitivity to detect low concentrations of gas, (ii) rapid response, (iii) reversible operation, (iv) good selectivity to different gases of interest, (v) low-manufacturing cost, (vi) stable operation over multiple cycles of usage, and (vii) low power consumption during the operation. Inkjet printing (IJP) is a promising route towards achieving the above desired gas sensor characteristics. IJP provides several advantages over other deposition techniques, such as dip-coating, spray coating, and electrophoretic deposition [56,57,58]. With inkjet printing, the process is rapid as no prefabricated masks or templates are required, and the cost of printing is low. Inkjet printing is a drop-on-demand process with five stages: drop ejection, drop flight, drop spreading, and drop solidification [59,60]. The feature resolution depends on drop volume, placement accuracy, and substrate-ink interaction. Droplet resolution is characterized by the size, shape, and volume of the drops affected by the nozzle size, fluid viscosity, and surface tension [56]. Viscosity, particle size, and solvent system of the ink are critical parameters for inkjet printing. Inkjet printing provides the advantages of rapid prototyping and on-demand digital printing in areas only where the material needs to be deposited. Constraints arise when dealing with the viscosity of the inks and particle size/concentration. Higher boiling point temperature solvents are also preferred when using an inkjet printer to avoid droplet jetting inconsistencies and coffee staining effects. When using water-based inks, tuning the viscosity and modifying the substrate surface energy (adding water-soluble sacrificial layer or oxygen plasma) can aid in obtaining higher resolution features [61,62]. Moreover, multiple layers can be printed with ease and the deposition of the material can be controlled with great precision. There is a great deal of research reported on inkjet printing using CNT and graphene for gas/vapor sensor applications, which we will further discuss.

In this paper, we discuss the recent developments in the area of inkjet-printed gas sensors using graphene and carbon nanotubes. The outline of the paper is as follows. First, in Section 1, we provide a brief overview of graphene and CNT nanomaterials along with the introduction to inkjet printing technique. In Section 2, we provide an overview of the recent experimental demonstrations in the area of inkjet-printed graphene-based gas sensors. In Section 3, we discuss important developments in the field of inkjet -printed carbon nanotube-based sensors for gas detection with emphasis on the impact of device geometry, the role of substrate engineering as well as the importance of chemical functionalization for printed CNT-based sensors. Section 4 describes some of the newer developments such as plasma jet printing and aerosol jet printing for the fabrication of graphene- and CNT-based gas detectors. Section 5 discusses in detail the role of defects on the performance of graphene and CNT devices, and finally in Section 6, we summarize important conclusions and scope for future research.

## 2. Graphene-Based Gas Sensors

Graphene has gained much interest from researchers since 2004 due to its remarkable electrical, mechanical, and thermal properties [63,64,65]. A high mobility, near-ballistic transport and stability at room temperature make graphene an ideal material for sensing applications, particularly gas/vapor detection [66]. Thus, graphene-based gas sensing device development has increased exponentially, and the number of published papers has sharply increased since 2007 [9,10,14,17,19,39,40,67,68,69,70,71,72,73,74,75,76,77,78,79]. In this section (Graphene-Based Gas Sensors), we will focus on inkjet printing of graphene-based gas/vapor sensor and their performance. The performance of a sensor is measured by its sensitivity, limit of detection, response time, recovery time and selectivity. Table 1 summarizes the sensing performance of recent reports on inkjet-printed graphene-based sensors for gas/vapor detection at room temperature.

**Figure 1:**
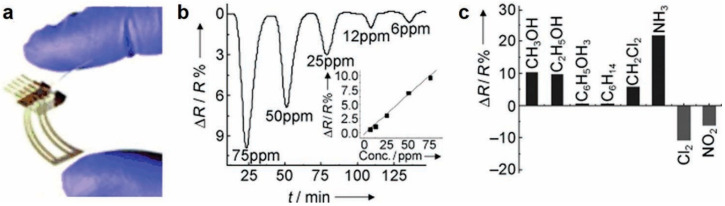
Flexisense, inkjet-printed graphene oxide and reduced graphene oxide for gas and vapor concentration detection [38]. (**a**) All-organic rGO-based flexible chemiresistor; (**b**) Resistance change versus time plot when the sensor was exposed to Cl_2_ vapor; (**c**) Change in resistance with exposed to other vapor; Reproduced with permission from John Wiley and Sons

Inkjet printing of an all-organic rGO-based chemiresistor to detect chemical vapors in the parts per million (ppm) to parts per billion (ppb) range at room temperature was first reported by Dua et al. [38]. The rGO ink was obtained by liquid phase exfoliation of graphite and dispersing the resulting flakes in aqueous surfactant solution. Furthermore, the exfoliated graphite oxide was reduced by a green chemistry alternative, ascorbic acid (vitamin C), than using aggressive reducing agents such as hydrazine. The lower number of covalently linked C-N species observed in X-ray photoelectron (XPS) spectra of rGO films makes it evident that ascorbic acid is an effective reducing agent compared to hydrazine. The rGO dispersion was inkjet printed with controlled uniformity of the sensing layers over a 3M overhead transparency PET film, seen in Figure 1a. A plot for resistance versus time when the sensor was exposed to Cl_2_ vapor is seen in Figure 1b with the signal response consistent with the photodesorption of the absorbed gases upon UV irradiation. The sensor shows a notable response to various aggressive vapors in a 100 ppm to 500 ppb concentration range and gas in a 10 ppm to 12 ppm concentration range, all at room temperature (Figure 1c). This work demonstrated that the use of very thin films shows a fast signal response and recovery compared to large films with a slow response/recovery time (minutes) for the inkjet-printed rGO-based gas/vapor sensors.

**Figure 2:**
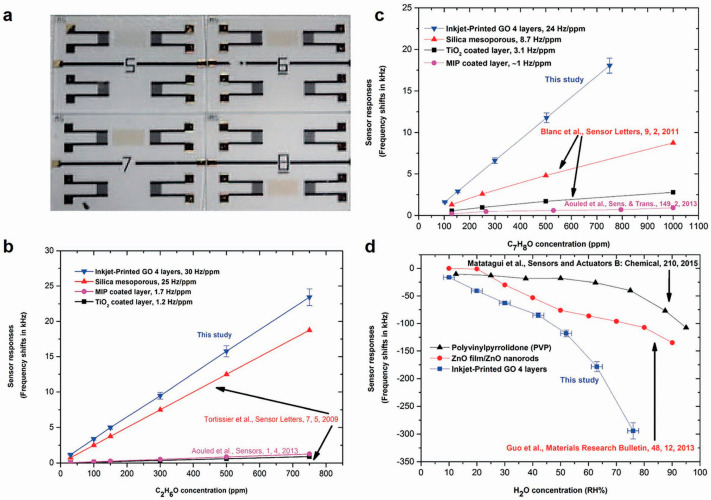
(**a**) Inkjet-printed graphene oxide on LOVE wave device. (**b**–**d**) Ethanol (C_2_H_6_O), toluene (C_7_H_8_) and H_2_O responses respectively, of different sensing layers (GO, ZnO film/ZnO nanorods and PVP) [85]. Reproduced with permission from IEEE.

Building on Dua et al.’s work, Nikolaou et al. reported inkjet printing GO layers on Shear Horizontal Surface Acoustic Wave (SH-SAW), also known as a Love wave sensor, wherein the performance of this platform enhances the trace-gas detection [85]. The sensing mechanism for this high performing sensor is dependent on the changes in electronic gain and the phase of the surface-confined acoustic wave propagation. Figure 2a displays the inkjet-printed GO coating on Love wave devices with different numbers of inkjet-printed passes (from 1 to 4 printed passes, corresponding to 5–8 devices seen in Figure 2a). Figure 2b–d compare the responses of different sensing materials with respect to GO material. Ethanol, toluene and H_2_O all show higher response to GO than the other sensing materials studied, such as silica mesoporous, TiO_2_ and molecular imprinted polymer. The layer-by-layer study of GO sensing material with the Love wave sensing platform offers a stable and reproducible solution for various gas sensing applications.

**Figure 3:**
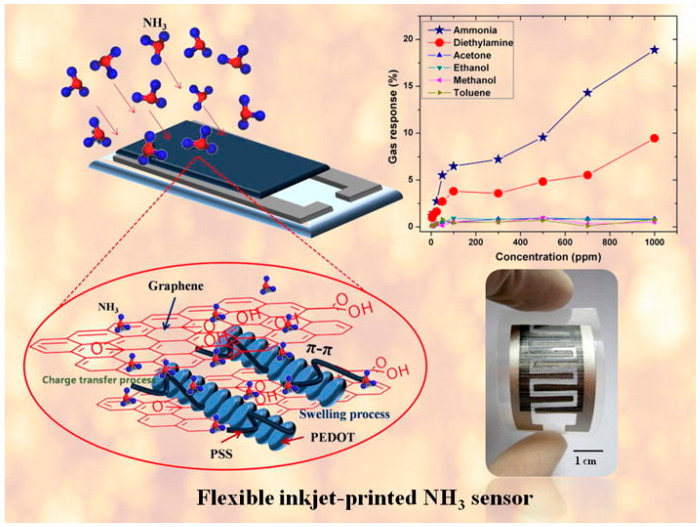
Flexible inkjet-printed GO/ PEDOT:PSS composite-based gas sensor for NH_3_ detection [84]. Reproduced with permission from Elsevier.

Moreover, Seekaew et al. reported a low cost and flexible inkjet-printed graphene/ PEDOT:PSS composite based gas sensor targeting ammonia [84]. Much like with Dua et al.′s work, the inkjet printing technique was used to achieve uniform layers over a large area. PEDOT:PSS, a conductive polymer was used with graphene to enhance sensor response and selectivity. Figure 3 captures the essence of the research in its entirety. The figure shows inkjet-printed graphene/ PEDOT:PSS sensing layer on top of the interdigitated screen-printed silver electrode on a flexible and transparent substrate. The figure also shows the excellent selectivity and sensing response time (S (%) = percentage change of the gas response) of ammonia gas to be in a range of 0.9–3.7% with a low concentration range of 25 to 1000 ppm at room temperature. With the addition of graphene to the PEDOT:PSS, the charge carrier concentration increased, and conduction channels of graphene enhanced the charge transport. The composite of graphene/ PEDOT:PSS based gas/vapor sensor showed much better performance than just PEDOT:PSS as the sensing material. The report suggests that a smooth surface of PEDOT:PSS film could lower the diffusion, and the short penetration depth of gas molecules may be the cause of a decrease in the sensor’s performance. Innovative composite materials and the low-cost fabrication technique of this gas sensor would provide a valuable solution to large-scale manufacturing of gas detectors.

**Figure 4:**
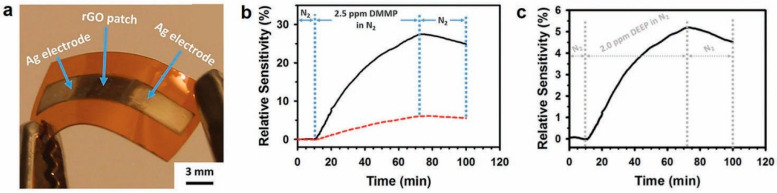
(**a**) Flexible gas sensor, with inkjet-printed reduced graphene oxide (rGO) and silver (Ag) electrodes on treated Kapton. (**b**,**c**) Relative sensitivity response to DMMP and DEEP in N_2_ gas at room temperature [86,87]. Reproduced with permission from Spring Nature and Royal Society of Chemistry.

With further fabrication enhancement, Fang et al. and their group reported on a flexible, bio-enabled, all inkjet-printed, rGO-based vapor sensor on modified Kapton substrate [86]. Figure 4a displays an optical image of the fully inkjet-printed rGO-based gas sensor. This work reported a sensing response of 2.5 ppm of dimethyl-methylphosphonate (DMMP) vapor in N_2_ carrier stream (Figure 4b). Over 1000 bend cycles, with varying radii of curvature, there were no detectable changes in the conductivity. Furthermore, this group demonstrated that modifying Kapton with polyelectrolyte multilayers (PEMs) significantly reduces the water contact angle and allows for good adhesion for the inkjet printing of the water-based rGO inks [87]. As a proof of concept, an inkjet-printed water-based rGO sensor on PEMs modified Kapton was fabricated to test the sensitivity of diethyl ethylphosphonate (DEEP) seen in Figure 4c. This novel approach offers a fully inkjet-printed, flexible, robust and lightweight solution for biosensing applications at room temperature. Herein, we summarized recent developments about inkjet-printed graphene-based gas sensors/vapor detection sensors.

## 3. Carbon Nanotubes-Based Gas Sensors

Carbon nanotubes (CNTs) and CNT composites are ideal candidates for gas sensing because of their extremely large surface area to volume ratio, making them intrinsically sensitive to any surface perturbations. Consequently, CNTs have been identified as being electrically sensitive to extremely small quantities of gases, electron acceptor and donor molecules such as humidity, oxygen, ammonia, nitrogen oxide and DMMP [2,7,43,88,89,90,91,92]. The sensitivity and selectivity can be further improved easily by suitable chemical functionalization of CNTs, e.g., oxygen-containing functional groups (-COOH and -OH) at the surface of CNTs lead to a much higher response than pristine CNTs [93,94]. In order to improve upon the sensitivity to specific gases, A Starr et al. fabricated an array of CNTFETs with different metal contacts and observed the specific transistor response for each FET as a function of metal contacts and target gas [95,96]. P. Bondavalli et al. demonstrated the use of SWCNT mats as channels for transistors in place of individual SWNTS fabricated with a dynamic spray gun technique to obtain highly controlled SWCNT densities [43]. Transistors were fabricated with different metals as S/D electrodes to demonstrate the difference in interaction of gases with the metal/SWCNTs junction on the Schottky barrier. However, unlike the classical Schottky barrier between metal and semiconductor, these contacts were unconventional because the SWCNTs were directly deposited on the metal without annealing. This model was originally presented by Yamada et al. for Au/SWCNT contacts [97,98]. Cui et al. studied the effects of adsorbed gases on the behavior of CNTFETs and showed that the gas molecule adsorption strongly influences the metal/SWCNT junction, changing the metal electrode work function and thus the Fermi level alignment [99]. These works were all important contributions in understanding the effects of gas adsorption on CNTFETs based gas sensors.

Kong et al. reported one of the earliest works on metal-decorated SWCNTs for H_2_ sensing [100]. In their work, Pd was deposited on individual SWCNTs by electron beam lithography, resulting in a measurable reduction in conductance upon exposure to ppm levels of H_2_ [100]. In order to obtain high performance from a SWCNT sensor, it is imperative to have a percolative network of semiconducting tubes, which are mainly responsible for changes in conductance due to the presence of adsorbed molecules [101]. Hybridization of CNTs with metal nanoparticles, metal oxides, and conducting polymers have shown significant performance improvements [102,103]. Several groups have successfully demonstrated integration of CNTs into inkjet-printed antenna systems for developing wireless gas sensing modules for detecting gases, such as ammonia and nitrogen dioxide [104,105]. A considerable amount of scientific reports and several excellent reviews on gas sensing properties of CNTs, multiwalled carbon nanotubes (MWNTs), and modified CNTs have been published [2,7,42,87]. The motivation for this section (Carbon Nanotube-Based Gas Sensors) is to provide the status of inkjet-printed carbon nanotube sensors in delivering ideally desired characteristics for gas sensing. In particular, the impacts of device geometry, substrate engineering and surface functionalization are discussed. Along with the existing state of the art, the goal is also to identify key future directions to deepen the fundamental understanding of chemical sensitivity of inkjet-printed CNTs and accelerate innovation towards devices/sensors utilizing these materials. For a broader, more general review on CNT gas sensors covering other fabrication methods, we direct the reader to the review paper by Meyyapan et al. [7]. Table 2 sums up the sensing performance of recent reports on inkjet-printed CNT-based sensors for gas/vapor detection at room temperature.

One of the earliest works on CNT-based chemical sensors was reported by Kong et al. for the detection of NH_3_ and NO_2_ [91]. The individual semiconducting SWNTs (S-SWNTs) were grown by CVD on SiO_2_/Si substrates and demonstrated molecular gating effects leading to the shifting of Fermi level of S-SWNTs, thereby modulating the resistance of the channel by orders of magnitude [91]. The chosen target gases resulted in two opposite electronic behaviors because of their chemical affinity: NO_2_ being an electron-acceptor gas (induced p-type doping of the SWNT) and NH_3_ being an electron-donor gas (induced n-type doping). The earliest inkjet-printed CNT gas sensor was reported by Jani Mäklin et al. for detecting H_2_S gas [109]. The active channel material was a carboxyl-functionalized nanotube film inkjet deposited between Ti/Pt based S/D electrodes with a PECVD-grown SiO_2_ layer as a gate dielectric. The sensor platform had an embedded heating circuit used to reset the sensor for rapid measurements. In this work, both a two-terminal resistive and three-terminal (p-type) Chem-FET device configuration were fabricated and tested. The Chem-FET sensor operated as p-channel transistor both for air and the H_2_S gas with increased/decreased channel conductivity at negative/positive gate bias. It was shown that H_2_S vapor induced an increased channel conductivity compared to the reference gas, demonstrating sensing capability of 100 ppm for these sensors. However, an order of magnitude higher change was observed for Chem-FET at low S/D bias and high positive gate bias compared to resistive sensors. The key mechanism was reported to be the modulation of junctions between semiconducting and metallic tubes in the network and Schottky barriers between CNTs and metal electrodes. This work highlights the importance of optimum device geometry for the improvement of inkjet-printed CNT gas sensors. The sensors in this work, however, did not recover reversibly after exposure to vapors was stopped and needed recovery achieved by heating the sensor up to 130 °C with the integrated Pt heating circuit for ~10 min.

**Figure 5:**
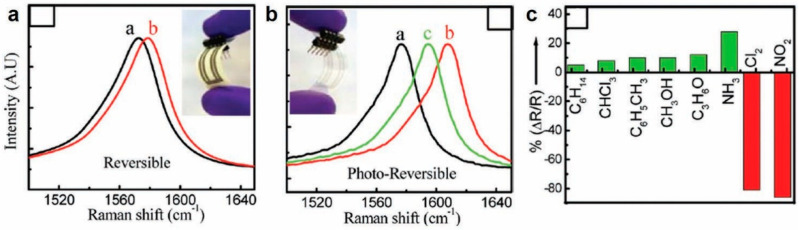
Raman shifts before (“**a**”, black) and after (“**b**”, red) exposure to 100 ppm Cl_2_ vapor for (**a**) inkjet-printed CNT/PET, where the shift is partially reversible upon photoirradiation (to “**c**”,green); (**b**) inkjet-printed CNT/paper, where the shift is reversible. (**c**) Selectivity plot for an inkjet-printed CNT/PET film, sensor exposed to saturated organic vapors, NH_3_ (100 ppm), NO_2_ (100 ppm), and Cl_2_ (100 ppm). Reproduced with permission from American Chemical Society [107].

The key advance in self-reversible sensors was made by Ammu et al. in demonstrating a reversible sensor for Cl_2_ and NO_2_ using inkjet-printed CNT films on cellulosic substrates (and plastics) that did not require thermal or photoirradiation for signal recovery [107]. In this work, NO_2_ was detected at concentrations as low as 125 ppb in ambient air for both PET and paper-based devices and the signal self-recovered upon removal of NO_2_. The physical mechanism behind this reversible response was attributed to the formation of a weak charge-transfer complex between NO_2_ and the CNTs that stops short of irreversible covalent bond formation. The behavior, however, was different for Cl_2_ vapors. Both PET and paper-based sensors demonstrated the detection capability of Cl_2_ vapor with concentrations as low as 500 ppb. For the PET substrate, the signal response did not recover spontaneously when Cl_2_ was removed, and it required additional photoirradiation for ~3 min. Even after this photoirradiation, the signal did not fully recover. However, a key finding was that for Cl_2_ detection, paper-based sensors showed reversible operation and self-recovered in ~7 min. This was further validated by an irreversible Raman shift for PET-based sensors, which only partially recovered with photoirradiation (Figure 5b) compared to paper-based sensors (Figure 5a) that show reversible Raman shift. The authors hypothesized that in the case of Cl_2_, with increased residence time, the vapors penetrate the interior of the CNT bundles and/or to the inter-bundle crossover points. This required additional external energy to recover signal or reset the sensor. Since the vapor residence time is significantly reduced on porous cellulosic substrates (as the vapor can desorb from all sides, as opposed to plastic substrates, where desorption is possible only from the top of the film), the paper-based sensors show reversible operation while PET-based sensors were irreversible. This work highlights the importance of substrate engineering for improved inkjet-printed CNT gas sensors. This work produced fully inkjet-printed and self-reversible sensors which were highly selective to target gases, as shown in Figure 5c.

**Figure 6:**
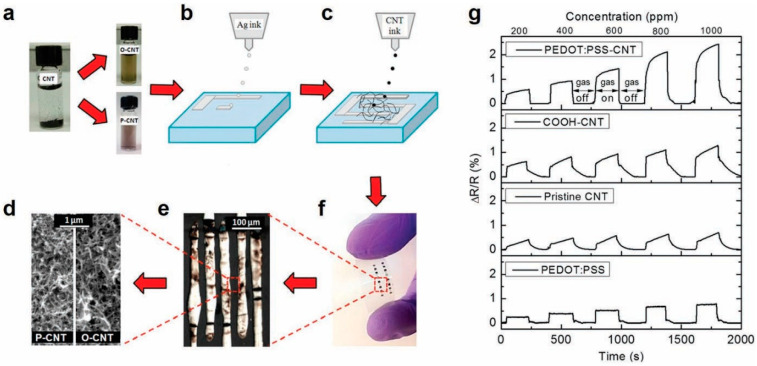
Fully printed and flexible CNTs based gas sensor: (**a**) CNTs’ functionalization with carboxylicacid (O-CNTs) and PEDOT:PSS (P-CNTs); (**b**) printing of Ag electrodes; (**c**) printing of CNTs; (**d**) photograph of the sensor on flexible substrate; (**e**) optical microscope image shows the printed silver interdigitated electrodes and (**f**) SEM image shows the printed carbon nanotubes. (**g**) Sensitivity of the printed ethanol vapor sensor (operated at 5V) with different CNTs functionalization methods and different gas concentrations. Reproduced with permission from Elsevier [53].

**Figure 7:**
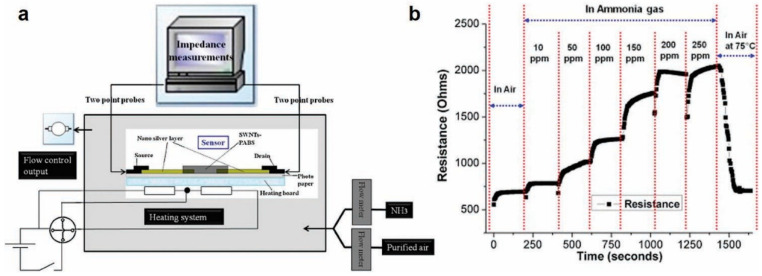
(**a**) Measurement setup for paper-based sensors with silver and inkjet-printed SWNT-PABS. (**b**) Resistance of paper-based sensor exposed to different concentrations of NH_3_. Reproduced with permission from Elsevier [54].

One promising direction to improve the sensitivity and selectivity of CNT-based sensors is in the functionalization of CNTs with different chemical groups, metal nanoparticles and organic molecules [94,102,103]. A recent experiment by Alshammari et al. showed the strong influence of functionalization on device performance [53]. In this work, three different CNT channels were investigated: (a) pristine CNTs with no functionalization; (b) CNTs functionalized with carboxylic acid (O-CNTs) and CNTs functionalized with conductive polymer PEDOT: PSS(P-CNTs). The method of fabrication and final inkjet-printed sensors are shown in Figure 6a–f. The sensitivity and the response time of the sensor for different functionalizations are shown in Figure 6g. Functionalization with carboxylic acid results in 1.7× enhancement in sensitivity compared to pristine CNTs while that with PEDOT:PSS results in 2.53× improvement in sensitivity. Similarly, Huang et al. demonstrated inkjet-printed NH_3_ gas sensors based on CNTs functionalized with poly (m-aminobenzene sulfonic acid) (PABS). Figure 7 shows the measurement setup and sensor response with a sensitivity of 10 ppm with these functionalized CNT based NH_3_ sensors on paper [54]. The sensor followed a step response, with a fast response time (~3 s), and was reversible and stable in outdoor environments for up to 3 months. Similarly, Timsorn et al. [55] demonstrated the impact of functionalization by fabricating a highly sensitive and extremely selective MWNTS-PEDOT:PSS-based sensor for formaldehyde in concentration range of 10–200 ppm at room temperature for food monitoring applications. The enhanced response in the nano-composite network-based sensors are the result of combining the sensing properties of both the constituent materials. The conducting polymers such as PEDOT PSS offer additional vapor attachment sites to the CNT network and also help in obtaining rapid response rates. This is because of the weak interaction between polymers and vapor molecules which can be easily desorbed upon exposure to air flow. Similarly, the performance enhancement in carboxyl-functionalized nanotubes is because oxygen is more electronegative than carbon, and attracts more electrons from electron donating vapors like ethanol, contributing to an increased change in the resistance of the sensor networks and improved sensitivity.

## 4. Role of Defects

### 4.1. Graphene-Based Sensors

Graphene has proven to be an excellent nanomaterial for application in chemical sensing, and the fundamental sensing performance is greatly affected by the role of defects that are induced by various fabrication processes. There have been several groups that have extensively studied the role of defects on the sensing mechanism of the graphene-based devices [18,19,20,27,31,36,117,118,119,120,121,122]. Defects such as film thickness, crystalline structure, porosity, wrinkles, grain boundaries, and external substrate defects all greatly affect the sensing performance of the sensor [19,20,22,75,76,123,124].

**Figure 8:**
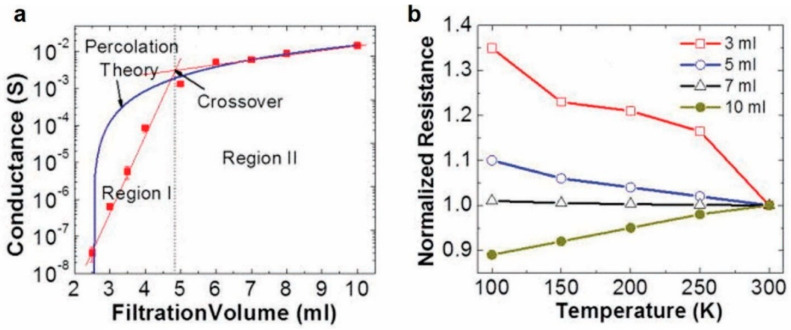
(**a**) Conductance (S) vs. filtration volume (mL) for the randomly stacked graphene flakes. (**b**) Normalized resistance vs. temperature (K) for various filtration volumes from 3 to 10 mL [18]. Reproduced with permission from AIP Publishing.

To explore these point and linear defects, Salehi-Khojin et al. demonstrated sensing performance of polycrystalline graphene ribbons compared to nearly pristine graphene [22]. CVD-fabricated graphene ribbons displayed higher sensitivity than of the pristine graphene due to the linear defects that are present, allowing for easy conduction pathways. Engineering linear defects and edges allows for improved sensitivity for graphene-based sensor. Moreover, Banerjee et al. and his team studied the electrochemical performance at the edge of the graphene nanopores fabricated by a TEM electron beam, isolated from the electrochemical contributions of the basal plane [23]. They observed that the electrochemical current densities were 3 times higher than those reported for CNTs and for pristine graphene. Manufacturing arrays of these nanopores could allow for superior sensing performance of gas sensors. Kumar et al.’s research showed that the defective CVD graphene-based gas sensors control the sensing characteristic of the device [19]. Moreover, their study showed that the defects on the SiO_2_ substrate were needed to modulate the electrical properties and are responsible for the sensing characteristics of the pristine graphene chemFETs. Another paper by Salehi-Khojin et al. analyzed the sensing performance of surfactant-assisted exfoliated graphene chemiresistor [18]. The sensing performance of the randomly stacked graphene flakes was characterized by controlling the filtration volume seen in Figure 8. The low filtration volume of the randomly stacked graphene flake sensor showed excellent sensitivity response, while the increase in filtration volume decreased in sensitivity as the electric transport regime switched from 2D electron hopping to phonon-limited (metallic) conduction. This sensor performed superiorly compared to other sensing materials such as polycrystalline graphene, graphene microribbon, and CNT-based chemical sensors. The review paper by Carbone et al. discussed that for graphene inks for inkjet printing, defects of different types are induced from the dispersing and stabilizing agents [125]. The dispersant and the stabilizing agents reduce the conductivity in the oxygenated species. Improvement regarding non-graphene components, such as using a proper conductivity polymer or even starch in the ink solution, tends to promote the performance of the overall sensor [75].

While the focus is to create defect-free nanomaterials, the next goal is to control/make defects in the materials (e.g., pores, edges, or replacing atoms) to self-repair, or engineer materials for catalytic or selectivity applications [117,126,127,128]. Zang et al. and their group demonstrated how defective graphene showed much stronger adsorption of different gas vapors than in pristine graphene [128]. Hajati et al. improved sensing in graphene material by gently inducing defects (reconstructed vacancies) in the lattice. This defect-controlled technique by Ga+ ion irradiation (~10^12^ ions cm^−2^) allows for improvements in transport properties in the graphene layer, in turn improving sensing and response time [129]. These studies showed that the defects induced by morphology, fabrication and different substrates play a significant role in sensing performance.

### 4.2. CNT-Based Sensors

**Figure 9:**
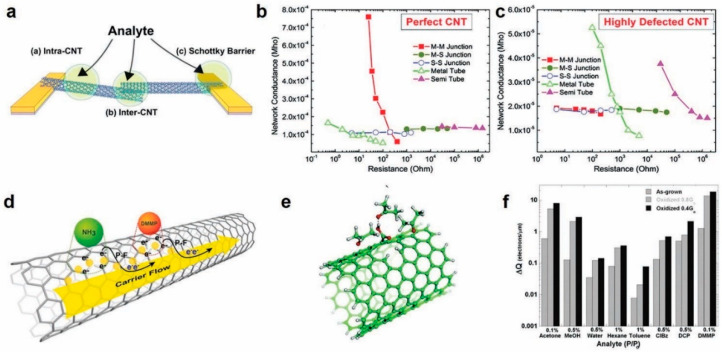
(**a**) Sensing mechanism in CNTs. Reproduced with permission from American Chemical Society [42]. (**b**) Calculations of the effects of changes in the components of the resistance on the overall resistance of the networks for perfect nanotubes and (**c**) defective nanotubes. Reproduced with permission from American Chemical Society [44]. (**d**) Detrapping mechanism of accumulated charges at the nanotube defects in PF regime. Reproduced with permission from AIP Publishing [131] (**e**) Clustering of acetone around the defect via intermolecular bonding. (**f**) Charge transfer between various analytes and the SWNT network as a function of oxidation. Reproduced with permission from American Chemical Society [132].

The pristine intrinsic properties of CNTs can be perturbed at various stages of the ink synthesis and printing process, for example during colloid formation, chemical functionalization, and oxidation. As such, a fundamental understanding of the impact of the defects on changes in CNT properties and corresponding change in sensing properties is imperative to designing CNT gas sensors. The sensing mechanism in CNTs can be explained according to interactions over three sections—along the length of tubes, at the junction between the tubes, or at the junction between the nanotubes and metal contacts, as shown in Figure 9a [42]. Fuhrer et al. proved that the contact resistance at the metal semiconducting junctions was two orders of magnitude larger than the resistance between two semiconducting or metallic SWCNTs, resulting in the current flowing preferably through either semiconducting or metallic tubes [130]. Khojin et al. did numerical computations and experiments to determine the change in the sensing mechanism of the chemiresistor upon addition of defects in the nanotubes [44]. They showed that in the case of perfect nanotubes, since the resistance of tubes is very small, the overall response of the chemiresistor mainly depends on the resistance changes at the junctions between the nanotubes as well as at the metal contacts to nanotubes junctions. Meanwhile, in the case of highly defective nanotubes, the resistance of the tubes is very high. Therefore, the overall sensor response is dominated by the resistance changes at the tubes themselves as compared to the other junctions. The key conclusion was that the main sensing mechanism is dependent on and changes according to the level of defects on the nanotubes, as shown in Figure 9b,c.

In another work, Khojin et al. showed that the conduction mechanism in the nanotubes is also related to the amount of defects [131]. They did measurements to show that at high electric fields, the Poole Frenkel mode of conduction dominates, wherein the electrons tunnel through the defects leading to an injection of trapped charge carries in the conduction band resulting in a higher response [131]. In other words, the Poole Frenkel regime effectively samples the defects, leading to higher sensitivity, as shown in Figure 9d. To understand and quantify the impact of defects on the overall sensitivity, Robinson et al. controllably introduced carboxylic acid sites through oxidation on the SWNTs (<2% of the total sites) and studied the impact on sensor response over a wide variety of gas vapors [132]. The samples that received more oxidation (0.4 G0) showed an enhanced response compared to samples with less oxidation (0.8 G0). An increase in both the capacitance and conductance response for a broad spectrum of analytes on SWNT was observed. The physical mechanism was attributed to defect sites serving as both low energy adsorption sites and nucleation sites for additional condensation of the gas species on CNT surface, as shown in Figure 9e–f. Once the analyte adsorbs at a defect site, charge transfer takes place between the analyte and CNTs, resulting in the resistance change. These works highlight a more general role of defects in sensing a wide variety of analytes and their implication on the design of printed gas sensors using carbon nanotubes.

## 5. Advanced Printing Techniques

In this section (Advanced Printing Techniques), we review the other state-of-the-art print modalities that are also being actively employed for printing gas sensors.

### 5.1. Aerosol Jet Printing

Aerosol jet printing (AJP) is another relatively new method of printing where the droplet size is much smaller than that of inkjet printing, resulting in finer features and higher resolution. AJP introduces new direct write capabilities with consistent deposition, allows a wider range of ink viscosities (1 to 1000 cP) and finer feature resolution (~10 µm). A typical AJP system consists of two modes of aerosolization: pneumatic and ultrasonic. The ultrasonic atomizer and the multi-axis positioning stage enables conformal printing on non-planar surfaces, such as on a golf ball. AJP allows for rapid integration when compared to other additive technologies [133]. However, AJP requires the tuning of several parameters, such as gas flow (or sheath gas N_2_), nozzle diameter, stage speed and substrate temperature, to achieve optimal print resolution. Therefore, it has been a challenge to print CNTs with AJP successfully. In an earlier work, Liu et al. successfully demonstrated Pt-functionalized SWNTs printed with AJP towards 40 ppm H_2_ detection without a coffee ring effect in the printed structures with N_2_ for carrier gas [116]. A recent work by Liang et al. further optimized the process and demonstrated high print resolution for the alignment of CNTs for flexible electronics applications using AJP [134].

In a novel technique, Zhou et al. demonstrated a highly efficient method of sorting semiconducting nanotubes by a new isoindigo-based copolymer to act as a channel material to construct aerosol jet-printed (with N_2_ carrier gas) thin film transistors (TFTs) on Si/SiO_2_ substrates [114]. TFTs based on these sorted semiconducting SWNTs showed superior device performance with high on/off ratios (10^6^:1) and mobility (up to 29.8 cm^2^·V^−1^·s^−1^) and small hysteresis. Gas sensors based on the above TFTs exhibited one of the best performances reported for NO_2_ sensors at room temperature with respect to sensitivity, stability and response rate.

In our research, we investigated power dissipation and electrical breakdown in aerosol jet-printed graphene (with N_2_ carrier gas) interconnects on Kapton, SiO_2_/Si, and Al_2_O_3_ substrates [135]. Our study indicated that the power dissipation in AJP graphene is dominated by the graphene interconnect morphology for high thermal conductivity substrates but can be limited by the substrate properties. Furthermore, our study showed that the porosity of the AJP-printed graphene induces a high thermal resistance of the graphene interconnects. An AJP printed (N_2_ carrier gas) metal oxide gas sensor reported by Cho et al. exhibits good sensitivity and fast response time (1.2 s) [136]. However, to our knowledge, there have not been any reports on AJP-printed graphene- or CNT-based gas sensors thus far.

### 5.2. Plasma Jet Printing

**Figure 10:**
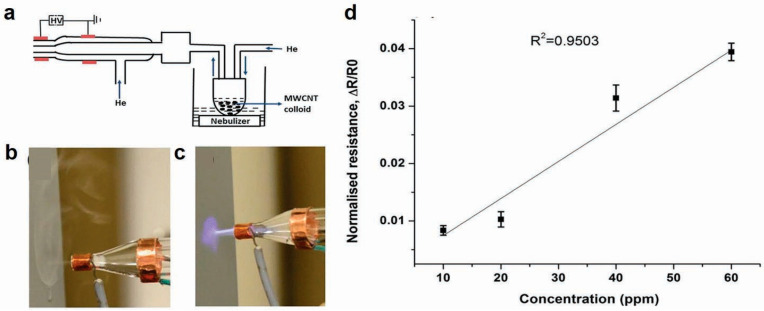
(**a**) Schematic of the atmospheric plasma jet. Photographs of aerosol flow with (**b**) no plasma and (**c**) plasma on. (**d**) MWCNTs on paper as a gas sensor exposed to ammonia in the range of 10–60 ppm. Reproduced with permission from AIP Publishing [106].

Although inkjet printing is a promising route towards printed CNT and graphene gas sensors, there are a few shortcomings including rigorous ink synthesis, nozzle clogging and the need for post-printing thermal treatment to remove dispersants (solvents, surfactants). Plasma jet printing (PJP) has shown promise in overcoming these challenges by enabling deposition of an aerosol at atmospheric pressure and at under 40 ℃ with no postprocessing required [106]. The setup for plasma jet printing is shown in Figure 10a. The printer consists of a quartz nozzle (diameter 5 mm) containing two copper electrodes (~2 cm apart) and connected to a high-voltage (1 to 15 kV AC) power supply [106]. A helium plasma is generated upon applying a potential between the electrodes. An ultrasonic nebulizer is used to atomize the colloidal material to create aerosol to be deposited. This aerosol is then carried to the print nozzle by a helium carrier gas into a quartz tube which contains the plasma. While the primary gas flow is at 2000 ccm, the secondary flow into the nebulization is at 50 ccm to aid in the transportation of the aerosol to the print head. The operation of the system with a fixed aerosol flow is shown in Figure 10b (plasma off) and Figure 10c (plasma on). This work used commercial MWCNTs and carboxyl functionalization to form the colloidal ink for plasma jet printing. The printed carbon nanotubes on paper showed a detection limit of 10 ppm towards NH_3_ (Figure 10d) and this work shows a promising direction for plasma jet printing for room temperature gas sensing. Moreover, PJP has shown potential to enhance conductivity in GO films by using a low-temperature He and H_2_ gas mixture to reduce a highly acidic GO suspension (pH < 2) in situ during deposition confirmed by XPS and NEXAFS (near-edge X-ray absorption fine structure spectroscopy). The reduction of carboxylic acid functional groups with the extended exposure to the plasma jet aids in yielding conductive GO patterns useful in gas sensing applications [137].

## 6. Outlook

The market for gas sensors is predicted to exceed USD 3 billion by 2027 [138]. There are innumerable applications for gas sensors ranging from environmental monitoring, wearable products, smart packaging of perishable food products, RFID tags and healthcare monitoring [110,139,140,141,142,143,144]. The motivation for making them flexible is to potentially increase the application areas of these sensors. Additive manufacturing techniques, such as inkjet printing, allow for large-scale, low-cost, portable sensor fabrication, without generating a lot of hazardous chemical waste as compared to traditional fabrication methods. Moreover, additive manufacturing allows for enhancing sustainability by using the resources efficiently and enable closed-loop material flows [145]. The inkjet printing method is less complex and provides higher throughput of devices than other traditional methods of fabricating sensors. The recent number of publications in the area of inkjet-printed graphene and carbon nanotube-based gas sensors shows an exponential rise, and thus needs further research.

## 7. Conclusions

Although CNT- and graphene-based gas sensors demonstrate great potential for next-generation printable and flexible sensing materials, several challenges remain before feature resolution and gas sensitivities can be compared to the conventional vacuum-based fabrication process. Many efforts to improve the inkjet printing process of CNTs and graphene for gas sensing applications are made by decorating CNTs or graphene with conductive oxides, polymers, or metals, improving the rheology of the ink, and substrate surface modification. With ongoing research in the area of ink synthesis, tuning printing process, and development of new printing methods, printed CNT- and graphene-based sensors will soon offer better control and resolution.

## Figures and Tables

**Table 1:** Printed graphene gas sensors.

Sensing Material	Printed Method	Target Gases	Detection Range/Sensitivity (Room-Temp)	Reference
Reduce Graphene Oxide	Inkjet	NO_2_ and several vapors	100 ppm to 500 ppb	[38]
Graphene/PEDOT-PSS	Inkjet	CO_2_	100 ppm/45 μOhm/ppm @ 30 ℃	[80]
Reduce Graphene Oxide	Inkjet	NH_3_	500 ppm	[81]
Reduce Graphene Oxide	Inkjet	NH_3_	10 ppm/2.80%	[77]
Reduce Graphene Oxide	Inkjet	NH_3_	500 ppm/6%	[82]
Graphene Oxide	Inkjet	NH_3_ and NO_2_	200–30 ppm, 150–2800 ppb	[83]
Graphene/PEDOT-PSS	Inkjet	NH_3_	5–1000 ppm	[84]
Graphene	Inkjet	NO_2_ and NH_3_	100 ppm/6.9% @ 250 ℃	[79]
Graphene Oxide	Inkjet	C_2_H_6_O, C_7_H_8_ and RH	30, 24, 2.4 Hz/ppm	[85]
Graphene Oxide	Inkjet	DMMP	2.5 ppm/27%	[86]
Reduced Graphene Oxide/Ag	Inkjet	DEEP	2.0 ppm/1%	[87]

**Table 2:** Printed carbon nanotube (CNT) gas sensors.

Sensing Material	Printing Method	Target Gas	Detection Range/Sensitivity (Room-Temp)	Reference
MWNT on paper	Plasma Jet	NH_3_	10–60 ppm/4%	[106]
SWNT on acid free paper	Inkjet	NO_2_, Cl_2_	NO_2_ 250 ppb, Cl_2_ 500 ppb	[107]
SWNT-PABS on paper	Inkjet	NH_3_	250 ppm	[54]
COOH/PEDOT:PSS-MWCNT on PET	Inkjet	C_2_H_5_OH	13 ppm	[53]
CNT	Inkjet	DMMP	10 ppm/20%	[77]
SWNT on Kapton	Inkjet	CO_2_	20,000 ppm	[105]
CNT on glass	Inkjet	NH_4_OH, Ethanol, Acetone	50–1000 ppm	[108,109]
SWNT-COOH on Si	Inkjet	H_2_S	100 ppm	[109]
Polymer(PVC/Cumene-PSMA/PSE/PVP)—CNTs on PEN	Inkjet	NH_3_	100 ppm/17%	[110,111]
PABS-SWCNT on paper	Inkjet	NH_3_	50 ppm	[112]
SWCNT on paper	Inkjet	NH_3_	-	[104]
Functionalized CNT on paper	Inkjet	NO_2_	30% at 10 ppm	[113]
SWCNT on Si/SiO_2_	Aerosol jet	NO_2_	96% at 60 ppm	[114]
SWCNT on Si/SiO_2_	Inkjet	NO_2_	5.7% at 10 ppb	[115]
MWCNTs/PEDOT: PSS	Inkjet	HCHO	30% at 10 ppm	[55]
Pt-SWCNTs	Aerosol jet	H_2_	1.5% at 40 ppm	[116]

## References

[1] Lee D.D. (2001). Environmental gas sensors. IEEE Sens. J..

[2] Yeow J.T.W., Wang Y. (2009). A review of carbon nanotubes-based gas sensors. J. Sens..

[3] Kohl D. (2001). Function and applications of gas sensors. J. Phys. D. Appl. Phys..

[4] Korotcenkov G. (2014). Handbook of Gas Sensor Materials.

[5] James D., Scott S.M., Ali Z., O’Hare W.T. (2005). Chemical sensors for electronic nose systems. Microchim. Acta.

[6] Yang S., Jiang C., Wei S. (2017). huai Gas sensing in 2D materials. Appl. Phys. Rev..

[7] Meyyappan M. (2016). Carbon Nanotube-Based Chemical Sensors. Small.

[8] Ryabtsev S.V., Shaposhnick A.V., Lukin A.N., Domashevskaya E.P. (1999). Application of semiconductor gas sensors for medical diagnostics. Sens. Actuat. B Chem..

[9] Wang T., Huang D., Yang Z., Xu S., He G., Li X., Hu N., Yin G., He D., Zhang L. (2016). A Review on Graphene-Based Gas/Vapor Sensors with Unique Properties and Potential Applications. Nano-Micro Lett..

[10] Basu S., Bhattacharyya P. (2012). Recent developments on graphene and graphene oxide based solid state gas sensors. Sens. Actuat. B Chem..

[11] Pumera M., Ambrosi A., Bonanni A., Chng E.L.K., Poh H.L., Lay E., Chng K., Ambrosi A., Bonanni A., Chng E.L.K. (2010). Graphene for electrochemical sensing and biosensing. TrAC Trends Anal. Chem..

[12] Chen D., Tang L., Li J., Geim A.K., Novoselov K.S., Brumfiel G., Sykes E.C.H., Geim A.K., Li D., Kaner R.B. (2010). Graphene-based materials in electrochemistry. Chem. Soc. Rev..

[13] Cinti S., Arduini F. (2017). Graphene-based screen-printed electrochemical (bio)sensors and their applications: Efforts and criticisms. Biosens. Bioelectron..

[14] Amin K.R., Bid A. (2014). Graphene as a sensor. Curr. Sci..

[15] Shao Y., Wang J., Wu H., Liu J., Aksay I.A., Lin Y. (2010). Graphene based electrochemical sensors and biosensors: A review. Electroanalysis.

[16] Meyyappan M., Koehne J.E., Han J.J.-W., Cress C.D., Schauerman C.M., Landi B.J., Messenger S.R., Raffaelle R.P., Walters R.J., Vitusevich S.A. (2015). Nanoelectronics and nanosensors for space exploration. MRS Bull..

[17] Schedin F., Geim A.K.K., Morozov S.V.V., Hill E.W., Blake P., Katsnelson M.I.I., Novoselov K.S.S. (2007). Detection of individual gas molecules adsorbed on graphene. Nat. Mater..

[18] Salehi-Khojin A., Estrada D., Lin K.Y., Ran K., Haasch R.T., Zuo J.M., Pop E., Masel R.I. (2012). Chemical sensors based on randomly stacked graphene flakes. Appl. Phys. Lett..

[19] Kumar B., Min K., Bashirzadeh M., Farimani A.B., Bae M.H., Estrada D., Kim Y.D., Yasaei P., Park Y.D., Pop E. (2013). The role of external defects in chemical sensing of graphene field-effect transistors. Nano Lett..

[20] Grosse K.L., Dorgan V.E., Estrada D., Wood J.D., Vlassiouk I., Eres G., Lyding J.W., King W.P., Pop E. (2014). Direct observation of resistive heating at graphene wrinkles and grain boundaries. Appl. Phys. Lett..

[21] Bae M.H., Ong Z.Y., Estrada D., Pop E. (2010). Imaging, simulation, and electrostatic control of power dissipation in graphene devices. Nano Lett..

[22] Salehi-Khojin A., Estrada D., Lin K.Y., Bae M.H., Xiong F., Pop E., Masel R.I. (2012). Polycrystalline graphene ribbons as chemiresistors. Adv. Mater..

[23] Banerjee S., Shim J., Rivera J., Jin X., Estrada D., Solovyeva V., You X., Pak J., Pop E., Aluru N. (2013). Electrochemistry at the edge of a single graphene layer in a nanopore. ACS Nano.

[24] Zhu Y., Murali S., Cai W., Li X., Suk J.W., Potts J.R., Ruoff R.S. (2010). Graphene and graphene oxide: Synthesis, properties, and applications. Adv. Mater..

[25] Stankovich S., Dikin D.A., Piner R.D., Kohlhaas K.A., Kleinhammes A., Jia Y., Wu Y., Nguyen S.B.T., Ruoff R.S. (2007). Synthesis of graphene-based nanosheets via chemical reduction of exfoliated graphite oxide. Carbon N. Y..

[26] Ciesielski A., Samorì P. (2014). Graphene via sonication assisted liquid-phase exfoliation. Chem. Soc. Rev..

[27] Paton K.R., Varrla E., Backes C., Smith R.J., Khan U., O’Neill A., Boland C., Lotya M., Istrate O.M., King P. (2014). Scalable production of large quantities of defect-free few-layer graphene by shear exfoliation in liquids. Nat. Mater..

[28] Bae S., Kim H., Lee Y., Xu X., Park J.S., Zheng Y., Balakrishnan J., Lei T., Ri Kim H., Song Y.I. (2010). Roll-to-roll production of 30-inch graphene films for transparent electrodes. Nat. Nanotechnol..

[29] Bao J., Norimatsu W., Iwata H., Matsuda K., Ito T., Kusunoki M. (2016). Synthesis of Freestanding Graphene on SiC by a Rapid-Cooling Technique. Phys. Rev. Lett..

[30] Wu Y., Lin Y., Bol A.A., Jenkins K.A., Xia F., Farmer D.B., Zhu Y., Avouris P. (2011). High-frequency, scaled graphene transistors on diamond-like carbon. Nature.

[31] Coleman J.N. (2013). Liquid exfoliation of defect-free graphene. Acc. Chem. Res..

[32] Dikin D.A., Stankovich S., Zimney E.J., Piner R.D., Dommett G.H.B., Evmenenko G., Nguyen S.T., Ruoff R.S. (2007). Preparation and characterization of graphene oxide paper. Nature.

[33] Moon I.K., Lee J., Ruoff R.S., Lee H. (2010). Reduced graphene oxide by chemical graphitization. Nat. Commun..

[34] Llobet E. (2013). Gas sensors using carbon nanomaterials: A review. Sens. Actuat. B Chem..

[35] Huang L., Wang Z., Zhang J., Pu J., Lin Y., Xu S., Shen L., Chen Q., Shi W. (2014). Fully Printed, Rapid-Response Sensors Based on Chemically Modified Graphene for Detecting NO _2_ at Room Temperature. ACS Appl. Mater. Interfaces.

[36] Vedala H., Sorescu D.C., Kotchey G.P., Star A. (2011). Chemical sensitivity of graphene edges decorated with metal nanoparticles. Nano Lett..

[37] Lu G., Ocola L.E., Chen J. (2009). Reduced graphene oxide for room-temperature gas sensors. Nanotechnology.

[38] Dua V., Surwade S.P.P., Ammu S., Agnihotra S.R.R., Jain S., Roberts K.E.E., Park S., Ruoff R.S.S., Manohar S.K.K. (2010). All-organic vapor sensor using inkjet-printed reduced graphene oxide. Angew. Chem. Int. Ed..

[39] Meng F.L., Guo Z., Huang X.J. (2015). Graphene-based hybrids for chemiresistive gas sensors. TrAC Trends Anal. Chem..

[40] Toda K., Furue R., Hayami S. (2015). Recent progress in applications of graphene oxide for gas sensing: A review. Anal. Chim. Acta.

[41] Eatemadi A., Daraee H., Karimkhanloo H., Kouhi M., Zarghami N., Akbarzadeh A., Abasi M., Hanifehpour Y., Joo S.W. (2014). Carbon nanotubes: Properties, synthesis, purification, and medical applications. Nanoscale Res. Lett..

[42] Schroeder V., Savagatrup S., He M., Lin S., Swager T.M. (2019). Carbon nanotube chemical sensors. Chem. Rev..

[43] Bondavalli P., Gorintin L., Feugnet G., Lehoucq G., Pribat D. (2014). Selective gas detection using CNTFET arrays fabricated using air-brush technique, with different metal as electrodes. Sens. Actuat. B Chem..

[44] Salehi-Khojin A., Khalili-Araghi F., Kuroda M.A., Lin K.Y., Leburton J.P., Masel R.I. (2011). On the sensing mechanism in carbon nanotube chemiresistors. ACS Nano.

[45] Arora N., Sharma N.N. (2014). Arc discharge synthesis of carbon nanotubes: Comprehensive review. Diam. Relat. Mater..

[46] Bonaccorso F., Bongiorno C., Fazio B., Gucciardi P.G., Maragò O.M., Morone A., Spinella C. (2007). Pulsed laser deposition of multiwalled carbon nanotubes thin films. Appl. Surf. Sci..

[47] Kumar M., Ando Y. (2010). Chemical vapor deposition of carbon nanotubes: A review on growth mechanism and mass production. J. Nanosci. Nanotechnol..

[48] Dai H. (2007). Nanotube Growth and Characterization. Carbon Nanotubes.

[49] Kharlamova M.V., Kramberger C., Yanagi K., Sauer M., Saito T., Pichler T. (2017). Separation of Nickelocene-Filled Single-Walled Carbon Nanotubes by Conductivity Type and Diameter. Phys. Status Solidi Basic Res..

[50] Chernov A.I., Obraztsova E.D. (2009). Density gradient ultra-centrifugation of arc produced single-wall carbon nanotubes. J. Nanoelectron. Optoelectron..

[51] Tortorich R.P., Choi J.-W. (2013). Inkjet Printing of Carbon Nanotubes. Nanomaterials.

[52] Byun K., Subbaraman H., Lin X., Xu X., Chen R.T. A 3μm Channel, Ink-Jet Printed CNT-TFT for Phased Array Antenna Applications. Proceedings of the 2013 Texas Symposium on Wireless and Microwave Circuits and Systems (WMCS).

[53] Alshammari A.S., Alenezi M.R., Lai K.T., Silva S.R.P. (2017). Inkjet printing of polymer functionalized CNT gas sensor with enhanced sensing properties. Mater. Lett..

[54] Huang L., Jiang P., Wang D., Luo Y., Li M., Lee H., Gerhardt R.A. (2014). A novel paper-based flexible ammonia gas sensor via silver and SWNT-PABS inkjet printing. Sens. Actuat. B Chem..

[55] Timsorn K., Wongchoosuk C. (1234). Inkjet printing of room-temperature gas sensors for identification of formalin contamination in squids. J. Mater. Sci. Mater. Electron..

[56] Singh M., Haverinen H.M., Dhagat P., Jabbour G.E. (2010). Inkjet printing-process and its applications. Adv. Mater..

[57] Deiner L.J., Reitz T.L. (2017). Inkjet and aerosol jet printing of electrochemical devices for energy conversion and storage. Adv. Eng. Mater..

[58] Sridhar A., Blaudeck T., Baumann R. (2009). Inkjet Printing as a Key Enabling Technology for Printed Electronics. Mater. Matters.

[59] Cummins G., Desmulliez M.P.Y. (2012). Inkjet printing of conductive materials: A review. Circuit World.

[60] Tekin E., Smith P.J., Schubert U.S. (2008). Inkjet printing as a deposition and patterning tool for polymers and inorganic particles. Soft Matter.

[61] Sun J., Li Y., Liu G., Chen S., Zhang Y., Chen C., Chu F., Song Y. (2020). Fabricating High-Resolution Metal Pattern with Inkjet Printed Water-Soluble Sacrificial Layer. ACS Appl. Mater. Interfaces.

[62] Nguyen P.Q.M., Yeo L.P., Lok B.K., Lam Y.C. (2014). Patterned surface with controllable wettability for inkjet printing of flexible printed electronics. ACS Appl. Mater. Interfaces.

[63] Novoselov K.S., Mishchenko A., Carvalho A., Neto A.H.C., Road O. (2016). 2D materials and van der Waals heterostructures. Science.

[64] Xia F., Wang H., Xiao D., Dubey M., Ramasubramaniam A. (2014). Two-dimensional material nanophotonics. Nat. Photonics.

[65] Mas-Ballesté R., Gómez-Navarro C., Gómez-Herrero J., Zamora F. (2011). 2D materials: To graphene and beyond. Nanoscale.

[66] Castro Neto A.H., Guinea F., Peres N.M.R., Novoselov K.S., Geim A.K. (2009). The electronic properties of graphene. Rev. Mod. Phys..

[67] Le T., Lakafosis V., Kim S., Cook B., Tentzeris M.M., Lin Z., Wong C. A novel graphene-based inkjet-printed WISP-enabled wireless gas sensor. Proceedings of the IEEE 2012 42nd European Microwave Conference.

[68] Cho B., Yoon J., Hahm M.G., Kim D.H., Kim A.R., Kahng Y.H., Park S.W., Lee Y.J., Park S.G., Kwon J.D. (2014). Graphene-based gas sensor: Metal decoration effect and application to a flexible device. J. Mater. Chem. C.

[69] Paul R.K., Badhulika S., Saucedo N.M., Mulchandani A. (2012). Graphene nanomesh as highly sensitive chemiresistor gas sensor. Anal. Chem..

[70] Latif U., Dickert F.L. (2015). Graphene hybrid materials in gas sensing applications. Sensors.

[71] Pearce R., Iakimov T., Andersson M., Hultman L., Spetz A.L., Yakimova R. (2011). Epitaxially grown graphene based gas sensors for ultra sensitive NO 2 detection. Sens. Actuat. B Chem..

[72] Choi W., Alwarappan S. (2018). Graphene-Based Biosensors and Gas Sensors. Graphene.

[73] Yoon H.J., Jun D.H., Yang J.H., Zhou Z., Yang S.S., Cheng M.M.C. (2011). Carbon dioxide gas sensor using a graphene sheet. Sens. Actuat. B Chem..

[74] Singh E., Meyyappan M., Nalwa H.S. (2017). Flexible Graphene-Based Wearable Gas and Chemical Sensors. ACS Appl. Mater. Interfaces.

[75] Peregrino P.P., Cavallari M.R., Fonseca F.J., Moreira S.G.C., Sales M.J.A., Paterno L.G. (2020). Starch-Mediated Immobilization, Photochemical Reduction, and Gas Sensitivity of Graphene Oxide Films. ACS Omega.

[76] Da Silva M.F.P., Souza E.J.P., Junior A.T.S., Cavallari M.R., Paterno L.G., Campos A.F.C., Fonseca F.J., Bernardi J.V.E., Landers R. (2020). Synthesis and characterization of GO-H_3_BO_3_ composite for improving single-sensor impedimetric olfaction. J. Mater. Sci. Mater. Electron..

[77] Hester J.G.D., Tentzeris M.M., Fang Y. Inkjet-printed, flexible, high performance, carbon nanomaterial based sensors for ammonia and DMMP gas detection. Proceedings of the 45th European Microwave Conference Proceedings, EuMC.

[78] Nguyen B.H., Nguyen V.H., Nguyen Bich H., Nguyen Van H. (2016). Promising applications of graphene and graphene-based nanostructures. Adv. Nat. Sci. Nanosci. Nanotechnol..

[79] Travan C., Bergmann A. (2019). NO2 and NH3 Sensing Characteristics of Inkjet Printing Graphene Gas Sensors. Sensors.

[80] Andò B., Baglio S., Di Pasquale G., Pollicino A., D’Agata S., Gugliuzzo C., Lombardo C., Re G. (2015). An inkjet printed CO2gas sensor. Procedia Eng..

[81] Bozzi M., Tentzeris M.M., Lakafosis V., Le T., Kim S., Vyas R., Georgiadis A., Cooper J., Cook B., Moro R. (2013). Inkjet-printed antennas, sensors and circuits on paper substrate. IET Microwaves Antennas Propag..

[82] Le T., Lakafosis V., Lin Z., Wong C.P., Tentzeris M.M. Inkjet-printed graphene-based wireless gas sensor modules. Proceedings of the 2012 IEEE 62nd Electronic Components and Technology Conference.

[83] Ricciardella F., Alfano B., Loffredo F., Villani F., Polichetti T., Miglietta M.L., Massera E., Di Francia G. Inkjet printed graphene-based chemi-resistors for gas detection in environmental conditions. Proceedings of the 2015 XVIII AISEM Annual Conference.

[84] Seekaew Y., Lokavee S., Phokharatkul D., Wisitsoraat A., Kerdcharoen T., Wongchoosuk C. (2014). Low-cost and flexible printed graphene–PEDOT:PSS gas sensor for ammonia detection. Org. Electron..

[85] Nikolaou I., Hallil H., Conedera V., Deligeorgis G., Dejous C., Rebiere D. (2016). Inkjet-Printed Graphene Oxide Thin Layers on Love Wave Devices for Humidity and Vapor Detection. IEEE Sens. J..

[86] Fang Y., Hester J.G.D., Su W., Chow J.H., Sitaraman S.K., Tentzeris M.M. (2016). A bio-enabled maximally mild layer-by-layer Kapton surface modification approach for the fabrication of all-inkjet-printed flexible electronic devices. Sci. Rep..

[87] Fang Y., Hester J.G.D., deGlee B.M., Tuan C.-C., Brooke P.D., Le T., Wong C., Tentzeris M.M., Sandhage K.H. (2016). A novel, facile, layer-by-layer substrate surface modification for the fabrication of all-inkjet-printed flexible electronic devices on Kapton. J. Mater. Chem. C.

[88] Kauffman D.R., Star A. (2008). Carbon nanotube gas and vapor sensors. Angew. Chemie Int. Ed..

[89] Zhang T., Mubeen S., Myung N.V., Deshusses M.A. (2008). Recent progress in carbon nanotube-based gas sensors. Nanotechnology.

[90] Ong K.G., Zeng K., Grimes C.A. (2002). A wireless, passive carbon nanotube-based gas sensor. IEEE Sens. J..

[91] Kong J., Franklin N.R., Zhou C., Chapline M.G., Peng S., Cho K., Dai H. (2000). Nanotube molecular wires as chemical sensors. Science.

[92] Li J., Lu Y., Ye Q., Cinke M., Han J., Meyyappan M. (2003). Carbon nanotube sensors for gas and organic vapor detection. Nano Lett..

[93] Fu D., Lim H., Shi Y., Dong X., Mhaisalkar S.G., Chen Y., Moochhala S., Li L.J. (2008). Differentiation of gas molecules using flexible and all-carbon nanotube devices. J. Phys. Chem. C.

[94] Sin M.L.Y., Chow G.C.T., Wong G.M.K., Li W.J., Leong P.H.W., Wong K.W. (2007). Ultralow-power alcohol vapor sensors using chemically functionalized multiwalled carbon nanotubes. IEEE Trans. Nanotechnol..

[95] Star A., Joshi V., Skarupo S., Thomas D., Gabriel J.C.P. (2006). Gas sensor array based on metal-decorated carbon nanotubes. J. Phys. Chem. B.

[96] Kauffman D.R., Star A. (2007). Chemically induced potential barriers at the carbon nanotube-metal nanoparticle interface. Nano Lett..

[97] Yamada T. (2004). Modeling of carbon nanotube Schottky barrier modulation under oxidizing conditions. Phys. Rev. B Condens. Matter Mater. Phys..

[98] Yamada T. (2006). Equivalent circuit model for carbon nanotube Schottky barrier: Influence of neutral polarized gas molecules. Appl. Phys. Lett..

[99] Cui X., Freitag M., Martel R., Brus L., Avouris P. (2003). Controlling energy-level alignments at carbon nanotube/Au contacts. Nano Lett..

[100] Kong J., Chapline M.G., Dai H. (2001). Functionalized carbon nanotubes for molecular hydrogen sensors. Adv. Mater..

[101] Kong J., Dai H. (2001). Full and modulated chemical gating of individual carbon nanotubes by organic amine compounds. J. Phys. Chem. B.

[102] Krishna Kumar M., Ramaprabhu S. (2006). Nanostructured Pt functionlized multiwalled carbon nanotube based hydrogen sensor. J. Phys. Chem. B.

[103] Wongchoosuk C., Wisitsoraat A., Phokharatkul D., Tuantranont A., Kerdcharoen T. (2010). Multi-Walled Carbon Nanotube-Doped Tungsten Oxide Thin Films for Hydrogen Gas Sensing. Sensors.

[104] Yang L., Zhang R., Staiculescu D., Wong C.P., Tentzeris M.M. (2009). A novel conformal RFID-enabled module utilizing inkjet-printed antennas and carbon nanotubes for gas-detection applications. IEEE Antennas Wirel. Propag. Lett..

[105] Vena A., Sydänheimo L., Tentzeris M.M., Ukkonen L. (2015). A fully inkjet-printed wireless and chipless sensor for CO2 and temperature detection. IEEE Sens. J..

[106] Gandhiraman R.P., Singh E., Diaz-Cartagena D.C., Nordlund D., Koehne J., Meyyappan M. (2016). Plasma jet printing for flexible substrates. Appl. Phys. Lett..

[107] Ammu S., Dua V., Agnihotra S.R., Surwade S.P., Phulgirkar A., Patel S., Manohar S.K. (2012). Flexible, all-organic chemiresistor for detecting chemically aggressive vapors. J. Am. Chem. Soc..

[108] Lorwongtragool P., Sowade E., Kerdcharoen T., Baumann R.R. All inkjet-printed chemical gas sensors based on CNT/polymer nanocomposites: Comparison between double printed layers and blended single layer. Proceedings of the 2012 9th International Conference on Electrical Engineering/Electronics, Computer, Telecommunications and Information Technology, ECTI-CON 2012.

[109] Mäklin J., Mustonen T., Halonen N., Tóth G., Kordás K., Vähäkangas J., Moilanen H., Kukovecz Á., Kónya Z., Haspel H. (2008). Inkjet printed resistive and chemical-FET carbon nanotube gas sensors. Phys. Status Solidi B.

[110] Lorwongtragool P., Sowade E., Watthanawisuth N., Baumann R.R., Kerdcharoen T. (2014). A novel wearable electronic nose for healthcare based on flexible printed chemical sensor array. Sensors.

[111] Lorwongtragool P., Sowade E., Dinh T.N., Kanoun O., Kerdcharoen T., Baumann R.R. Inkjet printing of chemiresistive sensors based on polymer and carbon nanotube networks. Proceedings of the International Multi-Conference on Systems, Signals and Devices, SSD 2012.

[112] Lee H., Shaker G., Naishadham K., Song X., McKinley M., Wagner B., Tentzeris M. (2011). Carbon-nanotube loaded antenna-based ammonia gas sensor. IEEE Trans. Microw. Theory Tech..

[113] Lin Z., Le T., Song X., Yao Y., Li Z., Moon K.S., Tentzeris M.M., Wong C.P. (2013). Preparation of water-based carbon nanotube inks and application in the inkjet printing of carbon nanotube gas sensors. J. Electron. Packag. Trans. ASME.

[114] Zhou C., Zhao J., Ye J., Tange M., Zhang X., Xu W., Zhang K., Okazaki T., Cui Z. (2016). Printed thin-film transistors and NO2 gas sensors based on sorted semiconducting carbon nanotubes by isoindigo-based copolymer. Carbon N. Y..

[115] Kim J., Yun J.H., Song J.W., Han C.S. (2009). The spontaneous metal-sitting structure on carbon nanotube arrays positioned by inkjet printing for wafer-scale production of high sensitive gas sensor units. Sens. Actuat. B Chem..

[116] Liu R., Ding H., Lin J., Shen F., Cui Z., Zhang T. (2012). Fabrication of platinum-decorated single-walled carbon nanotube based hydrogen sensors by aerosol jet printing. Nanotechnology.

[117] Pantelides S.T., Puzyrev Y., Tsetseris L., Wang B. (2012). Defects and doping and their role in functionalizing graphene. MRS Bull..

[118] Araujo P.T., Terrones M., Dresselhaus M.S. (2012). Defects and impurities in graphene-like materials. Mater. Today.

[119] Pak A.J., Paek E., Hwang G.S. (2014). Impact of Graphene Edges on Enhancing the Performance of Electrochemical Double Layer Capacitors. J. Phys. Chem. C.

[120] Park J., He G., Feenstra R.M., Li A.P. (2013). Atomic-scale mapping of thermoelectric power on graphene: Role of defects and boundaries. Nano Lett..

[121] Randviir E.P., Brownson D.A.C.C., Metters J.P., Kadara R.O., Banks C.E. (2014). The fabrication, characterisation and electrochemical investigation of screen-printed graphene electrodes. Phys. Chem. Chem. Phys..

[122] Varghese S.S., Lonkar S., Singh K.K., Swaminathan S., Abdala A. (2015). Recent advances in graphene based gas sensors. Sens. Actuat. B Chem..

[123] Ricciardella F., Vollebregt S., Polichetti T., Miscuglio M., Alfano B., Miglietta M.L., Massera E., Di Francia G., Sarro P.M. (2017). Effects of graphene defects on gas sensing properties towards NO_2_ detection. Nanoscale.

[124] Xu C., Xu B., Gu Y., Xiong Z., Sun J., Zhao X.S., Geim A.K., Novoselov K.S., Calizo I., Balandin A.A. (2013). Graphene-based electrodes for electrochemical energy storage. Energy Environ. Sci..

[125] Carbone M., Gorton L., Antiochia R. (2015). An overview of the latest graphene-based sensors for glucose detection: The effects of graphene defects. Electroanalysis.

[126] Ambrosi A., Bonanni A., Pumera M. (2011). Electrochemistry of folded graphene edges. Nanoscale.

[127] Vicarelli L., Heerema S.J., Dekker C., Zandbergen H.W. (2015). Controlling defects in graphene for optimizing the electrical properties of graphene nanodevices. ACS Nano.

[128] Zhang Y.H., Chen Y.B., Zhou K.G., Liu C.H., Zeng J., Zhang H.L., Peng Y. (2009). Improving gas sensing properties of graphene by introducing dopants and defects: A first-principles study. Nanotechnology.

[129] Hajati Y., Blom T., Jafri S.H.M., Haldar S., Bhandary S., Shoushtari M.Z., Eriksson O., Sanyal B., Leifer K. (2012). Improved gas sensing activity in structurally defected bilayer graphene. Nanotechnology.

[130] Fuhrer M.S., Nygård J., Shih L., Forero M., Yoon Y.G., Mazzoni M.S.C., Choi H.J., Ihm J., Louie S.G., Zettl A. (2000). Crossed nanotube junctions. Science.

[131] Salehi-Khojin A., Field C.R., Yeom J., Masel R.I. (2010). Sensitivity of nanotube chemical sensors at the onset of Poole-Frenkel conduction. Appl. Phys. Lett..

[132] Robinson J.A., Snow E.S., Bǎdescu Ş.C., Reinecke T.L., Perkins F.K. (2006). Role of defects in single-walled carbon nanotube chemical sensors. Nano Lett..

[133] Jabari E., Toyserkani E. (2015). Micro-scale aerosol-jet printing of graphene interconnects. Carbon N. Y..

[134] Goh G.L., Agarwala S., Yeong W.Y. (2019). Aerosol-Jet-Printed Preferentially Aligned Carbon Nanotube Twin-Lines for Printed Electronics. ACS Appl. Mater. Interfaces.

[135] Pandhi T., Kreit E., Aga R., Fujimoto K., Sharbati M.T., Khademi S., Chang A.N., Xiong F., Koehne J., Heckman E.M. (2018). Electrical Transport and Power Dissipation in Aerosol-Jet-Printed Graphene Interconnects. Sci. Rep..

[136] Cho Y.C., Elsayed M.Y., El-Gamal M.N. A Metal-Oxide Gas Sensor Based on an Aerosol Jet Printing Technology Featuring a One Second Response Time. Proceedings of the 2019 20th International Conference on Solid-State Sensors, Actuators and Microsystems and Eurosensors XXXIII, TRANSDUCERS 2019 and EUROSENSORS XXXIII.

[137] Dey A., Krishnamurthy S., Bowen J., Nordlund D., Meyyappan M., Gandhiraman R.P. (2018). Plasma Jet Printing and in Situ Reduction of Highly Acidic Graphene Oxide. ACS Nano.

[138] Chansin G., Pugh D. Environmental Gas Sensors 2017–2027. https://www.idtechex.com/en/research-report/environmental-gas-sensors-2017-2027/500.

[139] Gao W., Emaminejad S., Nyein H.Y.Y., Challa S., Chen K., Peck A., Fahad H.M., Ota H., Shiraki H., Kiriya D. (2016). Fully integrated wearable sensor arrays for multiplexed in situ perspiration analysis. Nature.

[140] Rose D.P., Ratterman M.E., Griffin D.K., Hou L., Kelley-Loughnane N., Naik R.R., Hagen J.A., Papautsky I., Heikenfeld J.C. (2015). Adhesive RFID sensor patch for monitoring of sweat electrolytes. IEEE Trans. Biomed. Eng..

[141] Hoe Y.Y.G., Johari B.H., Ju M., Kim S., Vaidyanathan K., Kang T.G. A microfluidic sensor for human hydration level monitoring. Proceedings of the 2011 Defense Science Research Conference and Expo, DSR 2011.

[142] Van Den Brand J., De Kok M., Koetse M., Cauwe M., Verplancke R., Bossuyt F., Jablonski M., Vanfleteren J. (2015). Flexible and stretchable electronics for wearable health devices. Solid. State. Electron..

[143] Tao X. (2005). Wearable Electronics and Photonics.

[144] Fan F.R., Tang W., Wang Z.L. (2016). Flexible nanogenerators for energy harvesting and self-powered electronics. Adv. Mater..

[145] Ford S., Despeisse M. (2016). Additive manufacturing and sustainability: An exploratory study of the advantages and challenges. J. Clean. Prod..

